# Atrial Fibrillation in Diabetes: Pathogenesis and Targeted Rhythm Control Strategies

**DOI:** 10.3390/cimb47070559

**Published:** 2025-07-17

**Authors:** Konstantinos Grigoriou, Paschalis Karakasis, Konstantinos Pamporis, Panagiotis Theofilis, Dimitrios Patoulias, Efstratios Karagiannidis, Barbara Fyntanidou, Antonios P. Antoniadis, Nikolaos Fragakis

**Affiliations:** 1 Department of Pharmacology, University of Athens, 11527 Goudi, Greece; dinosgrigoriou@gmail.com; 2 Second Department of Cardiology, Hippokration General Hospital, Aristotle University of Thessaloniki, 54642 Thessaloniki, Greece; aantoniadis@gmail.com (A.P.A.); fragakis.nikos@googlemail.com (N.F.); 3 First Cardiology Department, School of Medicine, Hippokration General Hospital, National and Kapodistrian University of Athens, 11527 Athens, Greece; konstantinospab@gmail.com (K.P.); panos.theofilis@hotmail.com (P.T.); 4 Second Propedeutic Department of Internal Medicine, Faculty of Medicine, School of Health Sciences Aristotle, University of Thessaloniki, 54642 Thessaloniki, Greece; dipatoulias@gmail.com; 5 Emergency Department, AHEPA University General Hospital, Aristotle University of Thessaloniki, 54642 Thessaloniki, Greece; stratoskarag@gmail.com (E.K.); bfyntan@yahoo.com (B.F.)

**Keywords:** diabetes mellitus, atrial fibrillation, rhythm control, atrial remodeling, atrial fibrosis, inflammation, SGLT2 inhibitors, GLP-1 receptor agonists

## Abstract

Diabetes mellitus and atrial fibrillation (AF) frequently coexist, creating a complex bidirectional relationship that exacerbates cardiovascular risk and challenges clinical management. Diabetes fosters a profibrotic, pro-inflammatory, and proarrhythmic atrial substrate through a constellation of pathophysiologic mechanisms, including metabolic remodeling, oxidative stress, mitochondrial dysfunction, ion channel dysregulation, and autonomic imbalance, thereby promoting AF initiation and progression. Conventional rhythm control strategies remain less effective in diabetic individuals, underscoring the need for innovative, substrate-targeted interventions. In this context, sodium–glucose cotransporter 2 (SGLT2) inhibitors and glucagon-like peptide-1 (GLP-1) receptor agonists have emerged as promising agents with pleiotropic antiarrhythmic properties, modulating fibrosis, inflammation, and mitochondrial integrity. Moreover, advances in anti-inflammatory, antifibrotic, and ion channel-modulating therapeutics, coupled with novel mitochondrial-targeted strategies, are reshaping the therapeutic landscape. Multi-omics approaches are further refining our understanding of diabetes-associated AF, facilitating precision medicine and biomarker-guided interventions. This review delineates the molecular nexus linking diabetes and AF, critically appraises emerging rhythm control strategies, and outlines translational avenues poised to advance individualized management in this high-risk population.

## 1. Introduction

Diabetes mellitus (DM) has emerged as a major public health concern, with a significant increase expected in the number of diagnosed individuals over the next two decades [1]. Beyond its well-established association with cardiovascular disease, DM has also been recognized as an independent risk factor for atrial fibrillation (AF) [2]. AF, the most prevalent sustained arrhythmia among adults worldwide, imposes a substantial burden on patients, healthcare systems, and the global economy, affecting more than 59 million individuals globally [3].

DM and AF frequently coexist, with DM affecting approximately 25% of individuals diagnosed with AF [4]. Emerging data indicate that individuals with type 2 DM (T2DM) are estimated to have a 38.6% likelihood of developing incidental AF [5]. Persistent DM accompanied by inadequate glycemic control further increases this risk by an additional 3% annually [6]. Moreover, type 1 diabetes mellitus (T1DM) has been linked to a moderate increase in AF risk among men (approximately 13%) and a substantially higher risk among women, reaching up to 50% [7]. The combination of DM and AF is associated with an impaired quality of life and a poorer prognosis characterized by increased mortality, a higher incidence of cardiovascular complications, and elevated healthcare costs [8,9]. The risk of developing AF increases in association with the severity of hyperglycemia, heightened glycemic variability, and prolonged duration of DM exposure [6,10,11].

AF represents the most common sustained cardiac arrhythmia encountered in contemporary clinical practice and is intricately linked to heightened morbidity, elevated all-cause and cardiovascular mortality, and a substantial increase in healthcare resource utilization and economic burden—regardless of symptomatic presentation [12,13]. Over the past decades, there has been significant progress in the antiarrhythmic treatment of AF, including both pharmacological and non-pharmacological approaches [14]. Despite technological advances in catheter ablation, the presence of DM, particularly when poorly controlled, is associated with a higher risk of post-ablation AF recurrence [15,16,17]. In terms of pharmacological treatment, several novel agents are now available for managing DM. Among these, sodium–glucose cotransporter 2 (SGLT2) inhibitors and glucagon-like peptide-1 (GLP-1) receptor agonists, owing to their multifaceted actions, have been proposed as valuable agents in the management of AF via their direct and predominantly indirect antiarrhythmic properties [18,19,20]

The growing prevalence of individuals with both AF and diabetes DM presents a complex public health challenge, primarily due to the complexities involved in managing these coexisting conditions [21]. This overlap exposes notable deficiencies in current treatment strategies, including clinicians’ reluctance to employ rhythm-control approaches in patients with DM, despite evidence that early rhythm control is associated with a reduced risk of DM-related complications and mortality [9,22,23]. Paradoxically, although DM patients are more likely to present with persistent or permanent AF, they are less frequently treated with rhythm-control therapies compared to euglycemic individuals [9]. Hence, there remains an unmet need for more effective, personalized, holistic, and molecularly targeted therapies to address the multifaceted clinical demands of patients affected by both DM and AF.

This review aims to summarize current evidence on the pathophysiologic link between AF and DM and explores emerging molecularly targeted therapies to better address the multifaceted clinical demands of patients with both conditions, with particular emphasis on rhythm-control strategies.

## 2. Methods

The present review was undertaken to critically synthesize contemporary evidence on the mechanistic interplay between diabetes mellitus and atrial fibrillation, with particular emphasis on pathophysiological substrates and emerging rhythm control strategies. A comprehensive literature search was conducted across PubMed, Scopus, and Web of Science databases up to June 2025. The search strategy employed combinations of controlled vocabulary and free-text terms, including but not limited to atrial fibrillation, diabetes mellitus, atrial remodeling, electrophysiological alterations, fibrosis, inflammation, GLP-1 receptor agonists, and SGLT2 inhibitors. Original experimental studies, clinical trials, meta-analyses, and high-quality mechanistic reviews published in English were screened for inclusion based on relevance to the scope of this study. Additional references were identified through manual citation tracking of pertinent articles.

## 3. Pathophysiologic Nexus Between Diabetes and Atrial Fibrillation

### 3.1. Metabolic Remodeling

**Figure 1:**
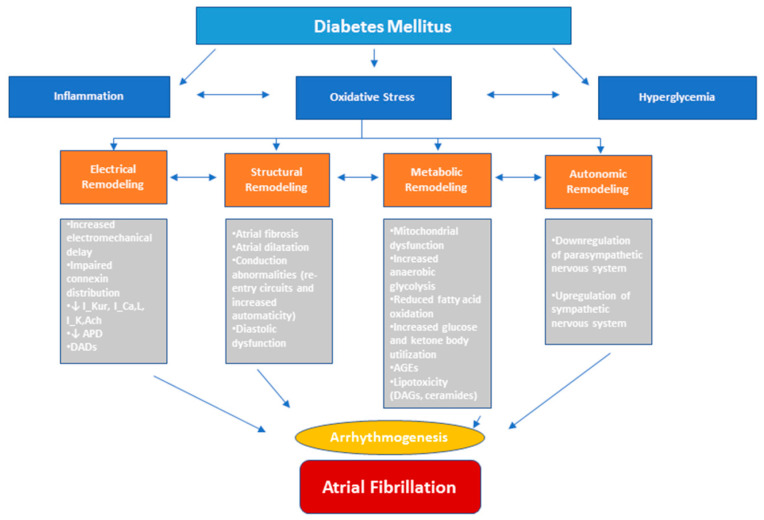
Pathophysiological drivers of diabetes mellitus (DM) in atrial fibrillation (AF). DM-associated AF arises from a convergence of multiple upstream mechanisms, including inflammation activation, oxidative stress, glucose fluctuations, and autonomic dysregulation. These processes contribute to the structural, electrical, metabolic, autonomic remodeling of the atrial myocardium, promoting both the initiation and perpetuation of AF. APD, action potential duration; DADs, delayed afterdepolarizations; AGEs, advanced glycation end-products; DAGs, diacylglycerols.

Beyond electrical and structural remodeling, emerging research highlights significant alterations in cellular metabolism associated with AF [24] (Figure 1). Known as metabolic remodeling, this process often precedes AF-related structural and electrophysiological alterations and involves changes in energy utilization within the atrial myocardium to meet the increased metabolic demands of AF. Over time, these changes can become maladaptive, impairing atrial function and contributing to both the onset and persistence of the arrhythmia [24,25].

Under normal conditions, the heart meets about 95% of its energy needs through mitochondrial oxidative phosphorylation, with fatty acid oxidation providing 40–60% of this ATP. The rest originates from pyruvate derived from glucose, lactate, and ketones. During AF, metabolic shifts occur, including increased anaerobic glycolysis, reduced glucose and fatty acid oxidation, and greater reliance on ketone metabolism [26]. Key molecular pathways implicated in these changes include Sirtuins3 (SIRT3)/AMP-activated protein kinase (AMPK) signaling, carnitine palmitoyltransferase-1 (CPT-1), acetyl-CoA carboxylase (ACC), peroxisome proliferator-activated receptor α (PPARα), and the downregulation of glucose transporter type 4 (GLUT4) [27,28,29].

T2DM promotes metabolic remodeling in AF by many ways such as altering energy substrate use, inducing mitochondrial dysfunction, and increasing oxidative stress and inflammation [24]. Additionally, T2DM causes lipotoxicity due to an imbalance between fatty acid uptake and oxidation, leading to the accumulation of toxic lipid intermediates such as diacylglycerols (DAGs) and ceramides [30]. These metabolites activate inflammatory signaling pathways—particularly through protein kinase C (PKC) and nuclear factor kappa B (NF-κB)—resulting in insulin resistance, apoptosis, and elevated pro-inflammatory cytokines like tumor necrosis factor (TNF), which contribute to atrial electrical and structural remodeling [31,32,33]. Furthermore, hyperglycemia promotes the formation of advanced glycation end-products (AGEs), which interact with their receptors (RAGE) to stimulate pro-fibrotic pathways, such as the upregulation of connective tissue growth factor (CTGF), causing atrial fibrosis in diabetic hearts [34,35].

Insulin resistance (IR) has been proposed as an independent risk factor for AF, even prior to the onset of diabetes, though clinical evidence remains mixed. Some studies, using HOMA-IR, show strong associations in individuals without T2DM [36], while others report no significant link [37,38]. However, animal studies consistently support a causal role for IR in developing AF, likely through molecular pathways involving increased transforming growth factor beta-1 (TGF-β1), oxidative stress, Rac1 activation, and disrupted calcium handling mediated by oxidized calcium/calmodulin-dependent protein kinase type II delta (CaMKIIδ), all contributing to atrial remodeling and arrhythmogenesis [39].

### 3.2. Inflammatory Signaling and Oxidative Stress

Inflammation, local and systemic, and oxidative stress are well-established factors contributing to the pathogenesis of AF in diabetic hearts. DM provokes systemic inflammation, characterized by elevated levels of inflammatory markers such as C-reactive protein (CRP), TNF-α, interleukin-6 (IL-6), and TGF-β1. These inflammatory mediators induce atrial anatomical and electrical remodeling [40,41]. Additionally, DM-related activation of NF-κB and NOD-like receptor protein 3 (NLRP3) inflammasome pathways further sustains inflammation within the atrial myocardium, enhancing the development of an AF-maintaining substrate [42,43]. Dysfunctional adipose tissues, particularly the epicardial fat layer, also contribute to the release of pro-inflammatory cytokines, while the release of anti-inflammatory adipokines diminishes. This imbalance leads to vascular endothelial damage, myocardial injury, oxidative stress, and increased thrombosis [44].

Oxidative stress, characterized by the enhanced production of reactive oxygen species (ROS), results from mitochondrial dysfunction and increased activation of nicotinamide adenine dinucleotide phosphate (NADPH) oxidase and xanthine oxidase [45]. Patients with DM exhibit both elevated levels of ROS and reduced activity of antioxidant enzymes such as glutathione peroxidase and superoxide dismutase, indicating a heightened burden of oxidative stress [40,42]. ROS contribute to the formation of a pro-arrhythmic substrate that facilitates the development and maintenance of AF through multiple molecular interrelated mechanisms. Specifically, ROS increase the oxidation of ryanodine receptor-2 (RyR2) and CaMKII, which promote arrhythmogenic Ca^2+^ leakage in atrial cardiomyocytes [45]. Furthermore, activation of the stress-responsive c-Jun N-terminal kinase isoform 2 (JNK2) facilitates RyR2-mediated diastolic sarcoplasmic reticulum (SR) Ca^2+^ leak via CaMKII, while independently enhancing sarco/endoplasmic reticulum Ca^2+^-ATPase (SERCA2) activity, which contributes to arrhythmogenesis [46,47]. In addition, persistent ROS activity activates the TGFβ1/Smad3 signaling pathway, which promotes fibrosis by upregulating fibrotic markers and enhancing the growth of fibroblasts [48]. Oxidative stress also contributes to electrical remodeling by activating PKC, which in turn leads to activation of the acetylcholine-regulated K^+^ current (I_K,Ach_) and shortening of action potential duration (APD), promoting the maintenance of AF [49]. Last, impaired nitroso–redox balance (increased ROS and reduced nitric oxide) may further contribute to arrhythmogenesis through the production of delayed afterdepolarizations (DADs) [42].

### 3.3. Fibrosis and Extracellular Matrix Remodeling

Atrial fibrosis plays a fundamental role in the development of atrial cardiomyopathy and contributes to both the genesis and progression of AF. It is characterized by the excessive accumulation of extracellular matrix (ECM) components, which enhances tissue stiffness and impairs normal cardiac structure and function. The pathophysiology underlying atrial fibrosis in AF is multifaceted and dynamic, involving stretch-induced fibroblast activation, fibrofatty infiltrations, localized and systemic inflammation, oxidative stress, and activation of coagulation pathways. In the context of AF, distinct patterns of fibrosis, such as reactive and replacement fibrosis, are observed and disrupt atrial tissue architecture. This structural remodeling promotes conduction abnormalities, facilitating re-entry circuits and increasing automaticity, both of which contribute to the persistence of AF [50,51].

AF-related atrial fibrosis is driven by complex molecular pathways that regulate ECM remodeling. One of the key mediators is TGF-β1, which activates the Smad signaling cascade, leading to enhanced transcription of fibrotic genes and significant dysregulations in the synthesis and degradation of collagen I, III, and IV [52,53]. The activated renin–angiotensin–aldosterone system (RAAS), primarily through angiotensin II, further enhances TGF-β1 expression and induces oxidative stress, both of which promote fibroblast activation and ECM deposition [53]. In this context, excessive ventricular myocardial fibrosis can lead to diastolic dysfunction, predisposing individuals with diabetes to abnormal ventricular filling and subsequent atrial enlargement [24]. Inflammatory cytokines, including TNF-α, IL-6, and IL-1β, also contribute to the process by enhancing pro-fibrotic signaling and subsequent fibroblast activation [54]. Additionally, the matrix metalloproteinase (MMP)/tissue inhibitor of metalloproteinases (TIMP) balance is impaired in AF, promoting fibrotic remodeling. Elevated levels of RECK, a membrane-anchored MMP inhibitor, further inhibit matrix degradation, exacerbating atrial structural remodeling [52].

DM creates a favorable environment that amplifies the fibrotic processes described above. In fact, DM is associated with myocardial fibrosis, independent of the presence of coronary artery disease or hypertension [53]. This association is mediated by enhancing key fibrosis-induced mechanisms, including oxidative stress and inflammation, activation of pro-fibrotic signaling pathways, accumulation of AGEs, and activation of RAAS [53]. These processes contribute to fibroblast activation and increased collagen ECM deposition, thereby leading to atrial structural remodeling and impaired myocardial function [50].

### 3.4. Electrophysiological Remodeling and Ion Channel Dysfunction

Electrophysiological remodeling constitutes a major substrate for diabetes-related AF. The majority of our current knowledge regarding electric and electromechanical alterations in diabetic hearts is derived from animal studies, owing to the limited amount of research conducted for this subject in the AF–DM population. Studies in diabetic animal models have quantified increases in inter-atrial conduction time (IACT), effective refractory period dispersion (AERPD), and prolongation of atrial APD [55,56]. These abnormal electrophysiological patterns are closely linked to changes in the expression and function of various ion channels in atrial myocytes (ion channel remodeling) which contribute to the increased AF susceptibility in DM [57]. Regarding sodium channels, studies have shown that sodium channel current (I_Na_) is reduced in the atrial myocyte tissue of Akita T1DM mice, primarily due to decreased expression of SCN5A mRNA and NaV1.5 protein [58]. In contrast, the late sodium current (I_Na,L_) is increased in diabetic mice [59]. Of note, this basal I_Na,L_ is mainly generated by the NaV1.5 isoform and is regulated by CaMKII [60]. Furthermore, diabetic-induced alterations of depolarizing L-type Ca^2+^ channels (I_Ca,L_) and repolarizing atrial-specific K^+^ channels, such as the Ca^2+^-activated small-conductance K^+^ current (I_SK_), the I_K,ACh_, and the ultrarapid delayed-rectifier K^+^ current (I_Kur_), can modify APD, thereby contributing to increased arrhythmogenicity [24].

Additional AF-induced electrophysiological features observed in diabetic hearts include reduced atrial conduction velocity, resulting from impaired connexin distribution [61] and decreased peak sodium current (Ι_Na,Peak_) [58], and an increased incidence of arrhythmogenic Ca^2+^ release events, driven by CaMKII activation and increased oxidative stress associated with DM [24]. Moreover, DM is frequently associated with impaired atrial excitation–contraction coupling and prolonged conduction times, leading to electromechanical delay (EMD), which is a recognized independent predictor of both new-onset and recurrent AF [62,63].

In summary, the electrophysiological remodeling in DM, encompassing ion channel alterations, prolonged APD, and reduced conduction velocity, can contribute to an increased susceptibility to AF.

### 3.5. Mitochondrial Dysfunction and Energetic Impairment

Mitochondrial dysfunction plays a critical role in both the initiation and maintenance of AF in diabetic hearts, driven by various underlying mechanisms [64,65]. As previously mentioned, diabetes-related mitochondrial impairment is associated with excessive ROS generation which contributes to mitochondrial DNA damage, altered gene transcription, enhanced activity of xanthine oxidase and NADPH oxidase, and activation of inflammatory pathways (NF-κB, caspase-1, and NLRP-3 inflammasome) [66,67]. Oxidative stress further promotes arrhythmogenesis by increasing the oxidation of ryanodine receptors and CaMKII, and by enhancing atrial fibrosis, an important factor of atrial structural remodeling [57].

In the context of DM, IR and subsequent hyperglycemia shift myocardial energy metabolism towards increased fatty acid oxidation and less towards carbohydrates, which is less efficient in terms of ATP production, thereby unable to meet the high energy demands of fibrillating atria [68]. This metabolic inflexibility further leads to elevated oxygen consumption and increased production of ROS derived from mitochondrial dysfunction. These changes cause significant anatomical and functional alterations in the atrial myocardium, such as fibrosis and dilation, which have proarrhythmic effects that facilitate the onset and progression of AF [69]. Furthermore, decreased ATP levels promote arrhythmogenesis by rapidly activating the sarcoplasmic ATP-sensitive potassium (K_ATP_) channels, which shorten APD and reduce action potential amplitude (APA) [70]. Moreover, mitochondrial biogenesis and function are compromised due to the downregulation of key regulatory proteins such as AMPK, PGC-1α, and SIRT1. This resulting energy shortfall disrupts ATP-dependent ion transport mechanisms, such as including SERCA2a and the Na^+^/K^+^-ATPase, thereby contributing to calcium dysregulation, electrical instability, and atrial structural changes. These metabolic and electrophysiological alterations synergically create a pro-arrhythmic substrate, facilitating both the development and persistence of AF in individuals with diabetes [71].

### 3.6. Autonomic Dysfunction

Autonomic imbalances, commonly observed in patients with DM, contribute to the development of atrial cardiomyopathy and increase the risk of AF [51]. In T2DM, cardiac autonomic neuropathy is characterized by increased sympathetic activity and reduced parasympathetic tone, as evidenced by findings from heart rate variability studies [72,73]. Parasympathetic stimulation can induce macro–reentrant circuits, whereas sympathetic activation may enhance abnormal automaticity and trigger activity. Moreover, autonomic dysfunction often precedes clinical diagnosis as blunted vagal responses and reduced acetylcholine release. These alterations impair atrial electrophysiology, thereby promoting the onset and maintenance of AF [74].

## 4. Emerging Molecular Targets for Rhythm Control in Diabetes-Associated AF

### 4.1. Anti-Inflammatory Pathways

Targeting inflammatory signaling has emerged as a promising therapeutic option, given the critical role of inflammation in the pathogenesis of AF among individuals with diabetes [75]. A key component of this inflammatory process is the activation of the NLRP3 inflammasome, observed in individuals with DM and paroxysmal, chronic, and postoperative AF [76]. Inhibition of the NLRP3 inflammasome can be achieved by therapeutic compounds targeting different signaling pathways [77]. Colchicine, a widely recognized and affordable anti-inflammatory drug, has been identified as a potential agent for the prevention of cardiovascular diseases by inhibiting the inflammatory axis. It exerts its anti-inflammatory effect by disrupting cytoskeletal functions in neutrophils and monocytes, inhibiting β-tubulin polymerization into microtubules, and suppressing chemotaxis [78]. Given that the spatial organization of the NLRP3 inflammasome is dependent on the integrity of the microtubule network, colchicine may serve as a promising therapeutic option for AF [79]. Incorporating insights from several trials suggest a general favorable trend of colchicine in preventing AF relative to placebo [77]. However, findings from the most recent randomized, placebo-controlled COP-AF trial showed that administration of colchicine in patients undergoing major thoracic surgery did not significantly reduce the incidence of post-operative AF [80]. Furthermore, several other compounds have been identified as direct inhibitors of the NLRP3 inflammasome and are currently under investigation for their antiarrhythmic effects. For instance, MCC950 reduced myocardial fibrosis, suppressed collagen production in animal models, and alleviated the risk of AF, while CY-09, RRx-001, JC-121, JC-124, and dapansutrile demonstrated favorable cardioprotective effects [77]. However, large-scale, randomized trials are necessary to evaluate their efficacy in preventing AF.

Targeting key products of the activated NLRP3 inflammasome, such as IL-1β, IL-18, and TNFα, may also be a promising approach for AF management, as these cytokines are upregulated in the serum of AF and post-operative AF patients and induce arrhythmogenesis [77]. Anakinra, an interleukin-1 receptor antagonist, and etanercept, a biologic TNF inhibitor, are potent anti-inflammatory drugs currently under clinical investigation for indications beyond cardiovascular diseases [79]. Canakinumab, a monoclonal IL-1β neutralizing antibody, has been proven to significantly reduce major cardiac events in patients with atherosclerosis (CANTOS trial) [81]. In 2020, the randomized, double-blind, placebo-controlled CONVERT-AF trial was conducted to evaluate the impact of canakinumab on preventing AF recurrence in patients with persistent AF undergoing electrical cardioversion to restore sinus rhythm. Although statistical significance for the primary endpoint was not achieved (*p* = 0.09), a promising trend was observed, with AF recurrence at 6 months occurring in 77% of patients in the placebo group and 36% in the canakinumab group [82].

Another category of agents with anti-inflammatory effects includes NF-κB inhibitors. Pinocembrin, an abundant flavonoid, has shown potential anti-AF properties in experimental MI animal model by mitigating atrial fibrosis, ameliorating autonomic and electrical remodeling, and suppressing inflammatory responses [83]. Additionally, experimental evidence suggests a critical role for the S100 protein family in the pathogenesis of various cardiovascular diseases [84]. Tasquinimod, an inhibitor of S100A8/A9 interaction and TLR4, improved the left ventricular systolic function and reduced the regulation of IL-1β, interferon-gamma (IFNγ), and TNFα in mice models, imposing as a potential antiarrhythmic agent [85]. Other targeting NLRP3 inflammasome-related pathways include the blockage of caspace-1, inflammasome component ASC, and NF-κB by compounds which are currently under investigation [77].

Beyond these pathways, another inflammatory mechanism that promotes AF is the infiltration of CCR2^+^ pro-inflammatory macrophages into the atrial myocardium, leading to pathological structural remodeling [86]. AF-related inflammation is associated with the recruitment of CCR2^+^ SPP1^+^ macrophages in the diseased human atrial myocardium, a process also seen in AF-vulnerable mice models [87]. SPP1^+^, also known as osteopontin, promotes atrial fibrosis and mediates signaling between immune cells. Studies in HOMER mice have shown that transplanting Spp1 bone marrow reduces AF maintenance and atrial fibrosis. Similar results were achieved using an antibody–siRNA conjugate to silence Spp1 in cardiac macrophages [88].

### 4.2. Anti-Fibrotic Signaling

Dissolving pre-existing collagen deposits in atrial tissue remains a significant therapeutic challenge. Current pharmacological approaches primarily aim to prevent the progression of atrial fibrosis associated with various cardiac conditions [89,90,91,92,93]. Targeting the RAAS through ACE inhibitors, angiotensin II receptor blockers (ARBs), or mineralocorticoid receptor antagonists (MRAs) may delay or prevent the development of atrial fibrosis [90,91,92,94]. Notably, finerenone, a nonsteroidal MRA, has been recently shown to reduce the risk of new-onset AF/AFL across the cardio–kidney–metabolic spectrum [95]. Several molecular pathways, including NF-κB, NADPH oxidase, lysyl oxidase homolog 2 (LOXL2), and the TGFβ1–SMAD2/3 signaling cascade, have been implicated in angiotensin II-induced atrial fibrosis [96,97,98,99]. Inhibiting these pathways may help reduce atrial fibrosis progression and lower the incidence of AF.

Additionally, endothelin 1 signaling has been shown to induce atrial remodeling in spontaneously hypertensive rats and exacerbate cardiac fibrosis in diabetic mice [100,101]. Macitentan, an endothelin receptor antagonist, decreased atrial endothelin 1 levels and prevented pacing-induced increases in pro-endothelin 1 mRNA. It also reduced atrial pro-inflammatory signaling, though it had minimal impact on calcium-regulating proteins, hypertrophy markers, and fibrosis indicators [100]. Protease-activated receptors 1 and 2 (PAR1 and PAR2) are also potential therapeutic targets for preventing atrial fibrosis in diabetic patients, but their effects are context-dependent, with evidence showing varied responses across cell types and experimental models, highlighting the need for further well-designed clinical trials to evaluate their efficacy [102,103,104,105].

Several microRNAs (miRNAs), such as miR-29, miR-30, miR-133, miR-21, and miR-590, regulate gene expression linked to cardiac fibrosis, with some inhibiting collagen production and others affecting TGFβ1 or SMAD3 expression [106]. miR-135b-5p and miR-138-5p influence glycosaminoglycan biosynthesis, though further validation is needed. Additionally, epigenetic mechanisms, like histone deacetylases and EZH2 expression, play roles in fibrosis regulation, suggesting new therapeutic opportunities for atrial fibrosis and AF [107,108,109,110,111,112,113,114,115]. Moreover, lutein, a naturally occurring carotenoid with metabolic benefits, also demonstrates potential anti-inflammatory, antioxidant, and antifibrotic effects in cardiac injury, although its therapeutic use is constrained by bioavailability challenges [116,117,118,119]. Furthermore, Wu et al. [120] investigated the role of the NLRP3 inflammasome-CASP1-galectin 3 pathway in AF and atrial remodeling, and evaluated the effects of glibenclamide, a hypoglycemic agent and NLRP3 inflammasome inhibitor, in diabetic rabbits. Their findings showed that glibenclamide treatment attenuated atrial fibrosis, restored epicardial conduction and conduction homogeneity, and decreased the inducibility of AF, all linked to the inhibition of the NLRP3 inflammasome [120].

### 4.3. Ion Channel and Calcium Handling Modulators

Given the limitations of classical antiarrhythmic agents, recent research in the field of AF has focused on identifying atrial-specific molecular targets, particularly ion channels and their upstream modulators, to achieve rhythm control with reduced proarrhythmic risk and fewer non-cardiac adverse effects.

Atrial electrical remodeling following hyperglycemia plays a pivotal role in the pathogenesis of AF, contributing to its onset and progression in DM [121]. This process is characterized by alterations in transmembrane ionic currents, involving the downregulation of L-type calcium current (I_Ca,L_) and the upregulation of inward rectifier potassium current (I_K1_) and I_K,ACh_. These changes synergistically shorten the atrial AP duration and refractory period, promoting reentry and ectopic activity [122,123,124,125]. Oxidative stress in DM further exacerbates these electrical alterations via redox-sensitive signaling pathways, notably those depended on CaMKII, a key modulator of metabolic and electrical remodeling in AF [24].

Among the most promising atrial-selective targets is I_Kur_ current, primarily mediated by the Kv1.5 channel. Kv1.5 is expressed almost exclusively in atrial tissue, making it an ideal target for atrial-selective blockade. Experimental compounds such as AVE0118, primarily an I_Kur_, I_to_, and I_K,ACh_ blocker, and XEN-D0101, a highly selective I_Kur_ blocker, have demonstrated efficacy in prolonging atrial refractory periods without affecting ventricular repolarization, thereby minimizing the risk of a proarrhythmic event [126,127,128,129,130,131,132,133,134].

Another important target is the I_Na,L_ current, which is enhanced in diabetic cardiomyocytes due to oxidative alterations of Na^+^ channels. This contributes to prolonged depolarization and Ca^2+^ overload. Ranolazine, a selective inhibitor of I_Na,L_, has demonstrated atrial-specific antiarrhythmic properties and is particularly promising in the context of diabetes-related electrical remodeling [135,136,137,138,139,140,141].

Another promising investigational pharmacological strategy for developing new anti-AF agents involves targeting gap junctions—clusters of ion channels that facilitate intercellular communication and connectivity. Each gap junction is composed of two hemichannels (connexons), and each connexon consists of six connexin proteins. Human atria particularly express connexin 40 and connexin 43 [142,143]. Under pathological conditions such as ischemia and diabetes-induced fibrosis, gap junction channels become dysfunctional, increasing the risk of arrhythmogenesis [144,145]. This dysfunction is characterized by reduced conduction and shortened APD, leading to greater dispersion of the refractory period [144]. Several selective gap junction enhancer peptides—such as AAP10, ZP123, and GAP-134—have been synthesized to regulate gap junction conduction, but their clinical efficacy remains to be fully investigated [146].

NCX has been identified as a potential therapeutic target for the prevention of AF, as its excessive reverse mode may promote arrhythmia by increasing the incidence of delayed afterdepolarizations. Experimental evidence suggests that NCX blockers, such as SEA-0400 and KBR7943, may prevent the onset and occurrence of AF. Despite these findings, large clinical studies are required to evaluate NCX blockers antiarrhythmic effects [147,148,149,150,151].

Furthermore, the ROS-CaMKII axis has garnered significant attention as a central arrhythmogenic molecular pathway in DM. Excessive ROS promotes the activation of CaMKII, which phosphorylates various ion channels including RyR2 and Na_v1.5, enhancing spontaneous Ca^2+^ release and I_Na,L_ [152]. In preclinical studies, inhibitors of CaMKII, such as KN-93 analogs, have shown promising results in suppressing AF by stabilizing calcium homeostasis and reducing I_Na,L_-mediated depolarizations [153,154]. In addition, abnormal sarcoplasmic reticulum (SR) Ca^2+^ leak via RyR2 has been implicated as one of the major mechanisms of afterdepolarizations and triggered activity in various AF models [154,155]. RyR2 stabilizers, such as JTV519 which enhances the binding affinity of FKBP12.6 to RyR2, may exert anti-AF effects by mitigating Ca^2+^ leak [156]. SERCA2a, another key calcium modulator, often shows diminished activity in the diabetic heart due to increased expression of phospholamban. The resulting calcium overload exacerbates delayed afterdepolarizations DADs and impairs atrial contractility. Targeting SERCA2a, through gene therapy using adenoviral-based delivery of Ca^2+^ binding proteins, may improve rhythm control [124].

### 4.4. Mitochondria-Targeted Therapeutics

Mitochondrial dysfunction and diabetes-associated AF have a close etiopathogenetic relationship. Diabetic atrial cardiomyocytes exhibit enhanced ROS production and mitochondrial hyperpolarization, leading to increased susceptibility to AF [157]. Several commonly used drugs, such as ACE inhibitors and statins, as well as nutraceuticals like CoQ10 and N-acetylcysteine, exert indirect effects on mitochondrial function and may offer a potential therapeutic approach for treating AF [158]. However, large-scale randomized trials are needed to confirm their efficacy and validate their role in AF prevention.

Novel antiarrhythmic approaches, such as mitochondria-targeted antioxidants, membrane stabilizers, and modulators of mitochondria biogenesis, represent exciting avenues but are still in early preclinical stages and face translational hurdles. Elamipretide is a mitochondria-targeted peptide that preserves the integrity of cardiolipin and improves the coupling of the electron transport chain. While some benefits have been observed in heart failure patients, its potential anti-arrhythmic effects against AF, remain to be established [159]. MitoQ, a mitochondria-targeted antioxidant, has demonstrated beneficial effects on the cardiovascular system, including the reduction in aortic stiffness and enhancement of endothelial function. It may also exert favorable effects on atrial remodeling by neutralizing ROS within the mitochondria [160]. Trimetazidine, a metabolic modulator, targets the respiratory chain by activating complex I and regulates the expression of factors involved in mitochondrial biogenesis. It is suggested that trimetazidine may exhibit antiarrhythmic properties by mitigating atrial structural remodeling, preventing the onset and maintenance of AF [161]. Moreover, KL1333, a novel NAD+ modulator, may exert its antiarrhythmic properties via enhancing the activity of AMPK/SIRT1/PGC-1a axis, which is crucial for maintaining mitochondrial metabolic homeostasis [162].

Gene-based approaches targeting key mitochondrial regulators such as PGC-1a, mitochondrial catalase (mCAT), and mitochondrial transcription factor A (TFAM) represent promising avenues for clinical translation [161]. Additionally, innovative technologies like lipid nanoparticle delivery systems and adenovirus vectors hold promise in addressing the arrhythmogenic substrate in diabetes-associated AF [50].

## 5. Modulation of AF Substrate by SGLT2 Inhibitors and GLP-1 Receptor Agonists

### 5.1. SGLT2 Inhibitors

SGLT2 inhibitors represent a relatively novel category of oral antidiabetic agents that function by inhibiting the renal reabsorption of sodium and filtered glucose. This action declines the renal threshold for glucose absorption in the proximal convoluted tubules, thereby causing glycosuria and natriuresis. These agents have demonstrated unique cardio–renal–metabolic benefits, with emerging evidence revealing their surprisingly significant benefits in various cardiovascular conditions, including AF onset or maintenance, regardless of glycemic status [20,163,164,165]. Indeed, SGLT2is, owing to their multifaceted pathophysiological actions, have emerged as valuable agents in the treatment of AF due to their direct and indirect antiarrhythmic properties [20].

Inflammation induces the development and progression of atrial structural and electrical remodeling, particularly in diabetes [20]. Emerging evidence from animal and human studies suggests that SGLT2is could be beneficial to prevent AF episodes by attenuating the inflammatory response [166,167]. SGLT2is suppress the release of circulating pro-inflammatory and inflammatory cytokines such as CRP, TNF-α, IL-6, MCP-1, TGF-β [167,168,169], regulate NO bioavailability [170], and modulate the activity of NLRP-3 inflammasome, thereby reducing the release of IL-1β and IL-18 [171]. Furthermore, SGLT2is enhance the reduction in EAT, a highly metabolically active tissue which contributes to the onset and progression of AF [172,173].

Mitochondrial dysfunction and oxidative stress are pivotal in atrial cardiomyopathy and AF progression across both individuals with and without diabetes [68]. ROS generated from mitochondrial dysfunction contribute to atrial fibrosis, dilation, and hypertrophy, exerting proarrhythmic effects and facilitating the onset and progression of AF [69,70]. Experimental animal studies have demonstrated that SGLT2is reduce ROS production, improve mitochondrial respiratory capacity, increase mitochondrial biogenesis, and regulate intracellular calcium handling, thereby leading to the reversal of atrial myopathy [174,175]. In addition, SGLT2is enhance cellular autophagy by activating key metabolic regulators, including AMPK, sirtuins (SIRT1, SIRT3, SIRT6), and PGC1-α, while concurrently suppressing the activity of mammalian target of rapamycin (mTOR) [176]. Furthermore, SGLT2is induce a metabolic shift from glucose to ketone bodies, thereby enhancing myocardial energy efficiency [20].

Atrial fibrosis plays a critical role in both the initiation and progression of AF. SGLT2is attenuate atrial fibrosis through multiple mechanisms. As mentioned above, these agents enhance mitochondrial function and reduce the production of ROS, thereby suppressing pro-fibrotic signaling pathways such as the TGF-β/SMAD axis [177]. In diabetic db/db mice, treatment with empagliflozin also led to the downregulation of pro-fibrotic proteins, including serum/glucocorticoid-regulated kinase 1 (SGK1) and the epithelial sodium channel (ENaC), resulting in reduced interstitial fibrosis in atrial tissue [178]. In addition, SGLT2 inhibitors exhibit anti-inflammatory properties by lowering circulating levels of cytokines like IL-6 and TNF-α involved in cardiac fibroblast activation and ECM remodeling [171,179,180]. Furthermore, their natriuretic and diuretic effects reduce atrial wall stretch and pressure, thereby alleviating mechanical stress-induced fibrosis [20,181].

Further, indirect antiarrhythmic mechanisms of SGLT2is include the stimulation of erythropoietin production and release, which enhance atrial oxygen delivery, the reduction in plasma uric acid levels, a known AF-risk factor, and the renal reabsorption of Mg^2+^ [20]. Moreover, SGLT2is enhance autonomic nervous system regulation by suppressing GPR41 in aortic, cardiac, and renal tissues, along with avoiding hypoglycemia and significant alterations in intravascular fluid volume [182,183].

Recent studies highlight the direct effects of SGLT2is on atrial electrophysiology. In a recent study, the acute treatment of increased single-dose dapagliflozin on human atrial cardiomyocytes suppressed action potential formation by directly inhibiting I_Na,peak_ and, to a lesser extent, I_to_. These findings indicate the acute class I antiarrhythmic effects of dapagliflozin [184]. Also, SGLT2is inhibit I_Na,L_ in cardiomyocytes by interacting with Nav1.5 sodium channels, thereby providing cardioprotective and antiarrhythmic effects [185]. SGLT2is further reduce intracellular Na^+^ by inhibiting sodium–hydrogen exchanger 1 (NHE-1), thereby reversing calcium overload [186]. Muller et al. showed that dapagliflozin directly interacts with human atrial K^+^ channels, and that the activation of K2P2.1 and K2P17.1 currents may contribute to its beneficial antiarrhythmic effects [187]. Regarding Ca^2+^ homeostasis, data from animal studies reveal that empagliflozin can modify Ca^2+^ regulation and Na^+^/hydrogen-exchanger currents, contributing to its antiarrhythmic properties [188]. Furthermore, empagliflozin reduces CaMKII activity and CaMKII-dependent sarcoplasmic reticulum Ca^2+^ leak, which improves myocardial calcium homeostasis and helps alleviate contractile dysfunction and arrhythmias [189]. Similarly, dapagliflozin contributes to inhibiting Ang II-induced ox-CaMKII upregulation, leading to beneficial modification of the electrical and structural atrial substrate [190].

A meta-analysis of 22 clinical trials involving 52,115 patients demonstrated that treatment with SGLT2 inhibitors significantly reduced the risk of AF (relative risk [RR]: 0.82, 95% CI: 0.70–0.96) and embolic stroke (RR: 0.32, 95% CI: 0.12–0.85), irrespective of baseline glycemic status [191]. Another meta-analysis of 22 trials assessing the incidence of AF and atrial flutter (AFL) in patients with T2DM and HF, SGLT2 inhibitors led to an 18% reduction in AF/AFL occurrence compared to the placebo [192]. Similar findings were reported in a post hoc analysis of the DECLARE-TIMI 58 trial which showed fewer episodes of AF in T2DM patients treated with dapagliflozin [193]. Furthermore, these agents reduced atrial tachyarrhythmias by 22% in patients with cardiac implantable electronic devices (CIEDs) [194].

### 5.2. GLP-1 Receptor Agonists

GLP-1 RAs are a well-established class of antidiabetic drugs that exert their glucose-lowering effects by stimulating glucose-dependent insulin release and suppressing glucagon secretion [195]. GLP-1 RAs have demonstrated remarkable cardioprotective effects in patients with T2DM, consistently reducing the risks of nonfatal myocardial infarction, stroke, and cardiovascular death [196,197]. However, these studies were not powered to investigate the effect of GLP-1 RA treatment on AF risk, although a metanalysis suggested potential antiarrhythmic benefits compared to other antidiabetic agents [198].

Experimental studies in animal models of DM have demonstrated a reduction in AF burden after treatment with GLP-1 RAs. In a canine model of pacing-induced AF, liraglutide reduced AF inducibility and improved electrophysiological parameters, including the shortening of the atrial effective refractory period and a reduction in conduction velocity compared to the placebo [199]. Consistent with these favorable findings, treatment with liraglutide in the db/db mouse model of T2DM reduced both AF susceptibility and duration by preventing atrial fibrosis and adverse atrial electrical remodeling. At a molecular level, this study showed that chronic treatment with liraglutide prevented the reductions in Ito current and I_Kur_ current, restored the steady-state inactivation of I_Na_ current, and normalized the expression of collagen-related genes Col1a and Col3a in atrial myocytes of db/db mice [200].

Emerging preclinical in vitro and in vivo data suggest that GLP-1 receptor agonists may exert antiarrhythmic effects through a range of cytoprotective molecular mechanisms. GLP-1 RAs directly counteract atrial fibrosis by modulating cardiac fibroblast activity through GLP-1 receptor activation. This action suppresses pro-fibrotic molecular pathways, including TGF-β and CTGF, and attenuates ECM deposition [201,202]. In addition, GLP-1 RAs significantly reduce systemic and local inflammation by attenuating macrophage infiltration and pro-inflammatory cytokine levels, such as TNF-α and IL-1β [203,204]. By promoting glucose uptake through p38MAPK-dependent increased translocation of GLUTs and enhancing fatty acid oxidation, these agents optimize cardiac metabolism, reducing metabolic stress that contributes to arrhythmogenesis [205]. GLP-1 RAs also bolster antioxidant and anti-apoptotic defenses mediated by activated PKA and Epac signaling pathways [206], diminishing oxidative stress and its fibrotic consequences [207]. Moreover, liraglutide has been shown to prevent IL-1β-induced impairments in mitochondrial homeostasis by enhancing the phosphorylation of AMPK and ACC, and upregulating PGC-1α, CPT1, and diacylglycerol acyltransferase 1 (DGAT1) [208]. Last, GLP-1 RAs alleviate indirectly cardiac hypertrophy via the angiotensin II/AT1R/ACE2 and AMPK/mTOR/p70S6K pathways, lessening mechanical strain on atrial tissue and further mitigating AF burden [209].

Clinical trials evaluating GLP-1 receptor agonists, such as the LEADER trial for liraglutide and the SUSTAIN-6 trial for semaglutide, demonstrated reductions in cardiovascular death, nonfatal myocardial infarction, and stroke. However, these studies were not designed to draw definitive conclusions about their impact on arrhythmic events, including AF [195,196]. While these medications have not been associated with an increased risk of AF as an adverse effect [210], comparative analyses suggest they are less effective than SGLT2 inhibitors in preventing new-onset AF in both the general population and high-risk populations, including older adults, women, and patients with cardiovascular disease or chronic kidney disease [211]. However, a recent meta-analysis showed that GLP-1 RAs are associated with a reduced risk of AF recurrence in patients undergoing AF ablation therapy [212].

## 6. Multi-Omics Approaches to Unravel Molecular Networks in Diabetes-Associated AF

Emerging advances in multi-omics strategies, integrating genomics, transcriptomics, epigenomics, proteomics, and metabolomics, have revolutionized our ability to dissect the molecular complexity of multifactorial diseases such as diabetes-associated AF. These integrative approaches enable a systems-level understanding that supports the identification of novel therapeutic targets and the development of precision medicine strategies.

A widely used method for identifying genetic variants linked to common diseases is the genome-wide association study (GWAS). This method enables researchers to examine and compare allele frequency, such as single-nucleotide polymorphisms (SNPs), between affected and unaffected individuals. GWAS have identified numerous AF- associated loci, like PITX2, RAP1A/KCND3, ZFHX3, HNF4G, and KCNN3, some of which regulate developmental, electrophysiological, contractile, and structural pathways [213,214,215]. However, a Mendelian randomization analysis involving over one million individuals of European ancestry found no clinically significant causal relationship between genetically determined T2DM, fasting blood glucose (FBG), or hemoglobin A1c (HbA1c) levels and the risk of developing AF [216]. This underscores the complexity of the relationship and the need for further investigation into the genomic underpinnings of diabetes-associated AF.

Transcriptomic analyses have significantly advanced our understanding of the molecular basis of diabetes-associated AF, revealing key alterations in gene expression that contribute to ion channel remodeling, inflammation, oxidative stress, thrombogenesis, and fibrosis [217]. A recent study using bioinformatics and network analysis identified more than 100 common differentially expressed genes (DEGs) in EAT from T2DM and AF datasets. Among them, CEBPZ, PAK1IP1, and BCCIP were revealed as central regulators in EAT, influencing the pathology of T2DM and AF [218]. Regarding microRNAs (miRNAs), a recent meta-analysis identified miR-4798, miR-133a, miR-150, miR-21, hsa-miR-4443, and miR-20a-5p as significantly associated with AF. The overall association between circulating miRNAs and AF showed an odds ratio (OR) of 2.51 [95% CI: 1.99–3.16; I^2^ = 99%], suggesting their potential as biomarkers for AF [219]. Furthermore, several miRNAs have been identified as potential regulators of gene expression associated with DM-related AF. For instance, upregulated miR-21 promotes fibrosis after MI by targeting TGF-β/Smad7 signaling pathway [220]. In contrast, overexpression of miR-133 and miR-30c reduces CTGF levels, thereby suppressing the production of collagens [221]. These miRNAs, as modulators of fibrosis, represent potential therapeutic targets for diabetes-associated AF.

Epigenetic regulatory mechanisms, such as DNA methylation, histone modifications, along with various non-coding RNAs, including miRNAs and circular RNAs (circRNAs), may play a role in the onset and progression of AF [222]. In DM, epigenomic research indicates that prolonged hyperglycemia leads to sustained changes in DNA methylation and histone modifications. These epigenetic alterations play a significant role in the onset and progression of diabetic complications [223,224]. Notably, class I histone deacetylase (HDAC) inhibitors have demonstrated anti-fibrotic effects in the heart, suggesting their potential as a novel therapeutic class for diabetes-associated AF [225].

Proteomics enables the detection of AF-related protein alterations, offering potential in the identification of diagnostic or prognostic biomarkers and novel therapeutic targets in individuals with diabetes. For instance, in a recent study, investigators examined proteomic differences between AF patients with and without T2DM. A total of 1548 proteins were identified, and 899 were quantified using label-free quantification (LFQ) analysis. This revealed 100 significantly dysregulated proteins—21 upregulated and 79 downregulated—in T2DM hearts. Notably, DPP9 and ATP1A2 were overexpressed, while INPPL1 and ARID5A were underexpressed [226].

Metabolomic studies have revealed significant metabolic disturbances in atrial AF, particularly in pathways related to energy metabolism, oxidative stress, and lipid utilization. In a notable study, Mayr et al. [227] performed integrated metabolomic and proteomic profiling of human atrial appendage tissues from patients with persistent AF. They reported a coordinated downregulation of enzymes involved in fatty acid oxidation and a concomitant upregulation of glycolytic enzymes, including triosephosphate isomerase and glyceraldehyde-3-phosphate dehydrogenase [227]. Given the established link between myocardial metabolism and ion channel expression, these metabolic shifts may contribute to arrhythmogenesis [228].

The integration of multi-omics data through network analysis enhances the understanding of complex molecular interactions in diabetes-associated AF and aids in identifying novel diagnostic markers and therapeutic targets.

## 7. Translational Perspective: Precision Rhythm Control in Individuals with Diabetes

The updated guidelines for the management of AF reinforce the urgent need to approach AF as a complex cardiovascular condition that requires disease prevention, risk-factor modification, as well as aggressive early rhythm control to prevent its progression. This therapeutic approach carries greater significance in the context of DM-associated AF, where clinicians must address multiple challenges [229].

**Figure 2:**
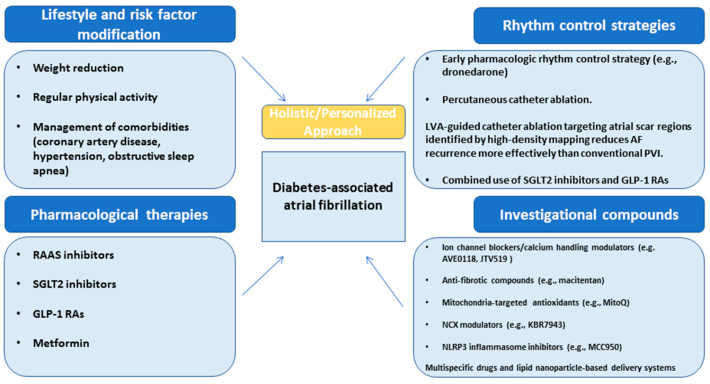
Therapeutic strategies span from upstream prevention to disease modification and downstream rhythm stabilization. Key upstream targets encompass lifestyle interventions, including sustained weight management and optimal treatment of comorbidities such as coronary artery disease, hypertension, and sleep apnea. Targeted approaches addressing neurohormonal and metabolic pathways—using agents such as RAAS inhibitors, SGLT2 inhibitors, and GLP-1 receptor agonists—address fundamental mechanisms of atrial remodeling. Treatment strategies focusing on NLRP3 inflammasome activation, mitochondrial dysfunction, specific atrial ion channels, and fibro-inflammatory signaling offer mechanistically precise avenues for reducing AF burden in diabetic patients. Rhythm control strategies, particularly early catheter ablation, employing novel techniques such as LVA-guided ablation, play a critical role. This comprehensive framework represents a paradigm shift toward substrate-focused, disease-modifying strategies in the management of DM-associated AF including RAAS, renin–angiotensin–aldosterone system; SGLT2, sodium–glucose cotransporter 2; GLP-1 RAs, glucagon-like peptide-1 receptor agonists; LVA, low-voltage area; PVI, pulmonary vein isolation.

As discussed in the current review, DM creates an arrhythmogenic substrate in the atrial myocardium via multiple pathophysiological mechanisms, leading to an increased risk of developing AF [5]. Hence, successful rhythm control requires a rather holistic approach that also includes effective glycemic control and proper management of comorbidities (Figure 2). It is known that DM often coexists with other known risk factors that promote the onset of AF, such as hypertension, coronary artery disease, obstructive sleep apnea, and obesity, all of which must be carefully managed through appropriate pharmacotherapy and lifestyle modifications in order to achieve rhythm control [229].

Glucose-lowering pharmacological interventions offer the potential to prevent atrial remodeling and reduce the risk of AF. However, not all antidiabetic drugs are equally effective in maintaining sinus rhythm. Metformin, the most commonly prescribed oral antidiabetic agent, mitigates AF burden through several mechanisms [230]. It attenuates oxidative stress by decreasing ROS production, exhibits anti-inflammatory properties, reducing circulating levels of cytokines IL-6 and TNF-α, improves calcium homeostasis, upregulates the expression of connexin-43 gap junction, restores current in small conductance Ca^2+^-activated K^+^ channels, activates AMPK, and modulates adiponectin signaling in the adipose tissue [231,232,233,234]. Thiazolidinediones (TZDs), another class of antidiabetic agents, have been shown to attenuate atrial fibrosis and reduce interatrial activation time, thereby potentially reducing AF vulnerability [235]. However, various clinical trials have yielded conflicted results regarding their overall efficacy in AF prevention [236,237,238]. Moreover, insulin use has been linked to an increased risk of new-onset AF in patients with T2DM, likely due to hypoglycemic episodes that trigger sympathetic activation and the more advanced stage of DM in these individuals [239]. Similarly, sulfonylureas are also associated with a significantly increased risk of new-onset AF [240,241]. Moving to more novel antidiabetic agents, a metanalysis of six clinical trials with 5250 DM patients showed that dipeptidyl peptidase-4 (DPP-4) inhibitors did not have any significant impact on AF risk, and in fact, increased the risk of atrial flutter [242]. SGLT2 inhibitors and GLP1 RAs are antidiabetic agents with potential antiarrhythmic effects. These drugs have demonstrated unique cardio–renal–metabolic benefits [243,244,245] with emerging evidence indicating their effectiveness in preventing AF incidence or recurrence, irrespective of baseline diabetes status [20,164,165].

Of note, while accumulating evidence suggests that certain glucose-lowering therapies may modulate AF risk, it is important to recognize that most large-scale cardiovascular outcome trials (CVOTs), to date, were not designed with arrhythmia endpoints as primary outcomes. Instead, AF incidence was typically captured as an exploratory or post hoc finding, limiting the strength of mechanistic and clinical inferences. For instance, the DECLARE-TIMI 58 trial observed fewer AF events in patients receiving dapagliflozin compared to placebo [193]; however, AF was not a prespecified endpoint, and systematic rhythm monitoring was lacking. In this context, a 2024 meta-analysis by Xu et al. [246], encompassing 32 randomized placebo-controlled trials and over 60,000 patients with T2DM, provides more targeted insight. The study demonstrated a statistically significant reduction in the risk of atrial arrhythmias (RR: 0.86; 95% CI: 0.74–0.99; *p* = 0.04) and atrial fibrillation/flutter (RR: 0.85; 95% CI: 0.74–0.99; *p* = 0.03) with SGLT2 inhibitor therapy—particularly among individuals with elevated cardiovascular risk and prolonged follow-up. Notably, the analysis did not show significant benefit for ventricular arrhythmias or bradyarrhythmias, underscoring the atrial-specific benefit of these agents [246]. Nonetheless, even this meta-analysis relies on heterogeneous trials with varying definitions, durations, and arrhythmia ascertainment methods. Therefore, ongoing prospective trials such as EMPA-AF (NCT04583813) and DAPA-AF (NCT05174052), which are explicitly designed to assess arrhythmic endpoints, will be instrumental in clarifying the rhythm-modifying potential of SGLT2 inhibitors and guiding evidence-based clinical decision-making in diabetes-associated AF.

To date, various studies have demonstrated that T2DM is associated with a reduced immediate success rate of cardioversion, as well as a decreased likelihood of maintaining sinus rhythm. In fact, T2DM has been identified as an independent risk factor for cardioversion failure within 30 days [247,248,249]. Similarly, animal studies suggest that antiarrhythmic drugs may be less effective in patients with T2DM; however, clinical evidence supporting this observation remains limited. Indeed, a single-center study reported no significant difference in cardioversion success rates between individuals with and without diabetes [250]. Regarding specific drug classes, T2DM did not appear to affect the efficacy of propafenone in achieving cardioversion, while dronedarone use was associated with a longer AF-free period [251,252].

Percutaneous catheter ablation is a well-established treatment for AF and has demonstrated effectiveness in patients with DM. Early intervention with this technique holds promise in attenuating the progression of atrial cardiomyopathy, provided the procedure is conducted in the early stages of the disease and achieves complete suppression of AF [253]. Some reports suggest that catheter ablation may be less effective in individuals with T2DM compared to those without, possibly due to the greater extent of atrial fibrosis commonly observed in this population [254]. Hence, targeting these fibrotic areas that contribute to an arrhythmogenic substrate may translate into improved survival free from AF recurrences. Indeed, low-voltage area (LVA)-guided ablation targeting LVAs, which represent atria scars, identified via high-density mapping, have shown to significantly reduced AF recurrences compared to conventional pulmonary vein isolation (PVI) [255]. Further, the use of late gadolinium enhancement cardiac magnetic resonance (LGE-CMR) to quantify atrial fibrosis may enhance the efficacy of fibrosis-guided ablation strategies, though further validation is required [50]. Of note, these approaches align with the growing emphasis on personalized, substrate-based therapeutic strategies in DM patients.

To further support the efficacy of catheter ablation in T2DM individuals, the combined use of SGLT2 inhibitors and GLP-1 RAs may synergistically attenuate AF burden by inhibiting the progression of DM-related atrial cardiomyopathy through complementary pathways. Both drug categories reduce oxidative stress and inflammation, mitigate neurohormonal activation, reduce pro-fibrotic signaling, provide metabolic efficiency, and improve endothelial function [20,164,165]. Furthermore, SGLT2 inhibitors’ diuretic effects [256] and GLP-1RAs’ impact on cardiac hypertrophy [257] reduce atrial wall stress, addressing mechanical drivers of atrial remodeling. By targeting multiple facets of atrial cardiomyopathy, the combination of these agents offers a comprehensive strategy for preventing post-ablation AF recurrence in individuals with diabetes. However, future clinical studies need to be conducted to determine whether the pathophysiological basis of their synergistic effects translates into a documented reduction in AF occurrence after catheter ablation.

Regarding cardiovascular implantable electronic devices (CIEDs), although their role in AF prevention may be limited, their rhythm-monitoring capabilities provide invaluable insights into the management of AF. In patients with CIEDs and an underlying AF substrate, a progressive decline in atrial electrogram amplitudes may indicate the development of atrial cardiomyopathy, imposing the importance of closer monitoring and strict managing of risk factors. Of note, DM has a high predictive ability of AF (OR 2.24, 95% CI 1.20–4.19, *p* = 0.011) for CIED-patients [258].

## 8. Future Directions and Knowledge Gaps

Despite significant advances in the management of diabetes-associated AF, there is an emerging need for effective treatment options capable of restoring and maintaining sinus rhythm in affected individuals. To achieve this goal, extensive research is currently focused on identifying previously unrecognized molecular mechanisms that can enhance our knowledge for the development of AF and permit the identification of novel therapeutic targets.

Classic antiarrhythmic drugs currently employed in AF-management are far from being ideal, and their already limited effectiveness in suppressing AF is further reduced in the presence of DM. Moreover, these agents raise safety concerns, and individuals with diabetes may face a heightened risk of adverse effects due to often concomitant QTc prolongation, coronary artery disease, and renal impairment [259].

Recent progress in elucidating the molecular pathophysiological links between DM and AF have paved the way for the development of targeted antiarrhythmic therapies (Table 1). These emerging strategies involve the modulation of previously overlooked ion channels, the inhibition of AF-related remodeling processes, and the targeting of specific molecular events implicated in AF generation. For these approaches to be applicable in clinical practice, they should exhibit high affinity for the atrial myocardium, while posing minimal proarrhythmic risk, avoiding non-cardiac adverse effects, supporting chronic administration, and preserving key homeostatic processes. To overcome these challenges, novel approaches, such as multispecific drugs and lipid nanoparticle-based delivery systems, represent exciting avenues; however, these technologies remain in early preclinical stages and face significant translational hurdles [50,260]. Furthermore, a broader use of omics technologies, such as genomics, transcriptomics, proteomics, and metabolomics, can uncover novel biomarkers and potential therapeutic targets, paving the way for personalized approaches tailored to each patient’s molecular profile [261]. For instance, it is conceivable that blood-based gene expression profiling could be used to screen candidates for catheter ablation, indicating those who are at lower risk of post-ablation AF.

In addition, SGLT2is and GLP-1RAs have emerged as potent therapeutic agents that extend their benefits beyond glycemic control. The ability of SGLT2is and GLP-1RAs to modulate multiple molecular pathways involved in atrial cardiomyopathy presents significant implications for the management of diabetes-associated AF. To definitively establish their efficacy in reducing the risk of AF in patients with diabetes, future research should prioritize on conducting large-scale RCTs with predefined AF endpoints. These studies should also align with the growing emphasis on personalized treatment and aim to address current heterogeneities by standardizing population characteristics, follow-up durations, and the specific SGLT2is and GLP-1RAs used. Furthermore, the precise role of these agents in preventing post-operative AF has yet to be determined. An additional key area for further investigation is whether SGLT2is and GLP-1 RAs can synergistically reduce the risk of diabetes-associated AF.

Regarding non-pharmacological antiarrhythmic strategies, catheter ablation appears to be less effective in diabetes-associated AF, primarily due to the presence of extensive atrial fibrosis. Identifying and targeting DM-induced fibrotic regions, responsible for creating an arrhythmogenic substrate, and performing ablation beyond the pulmonary veins, may improve outcomes and reduce AF recurrence in individuals with diabetes. These advancements underscore the importance of refining patient selection criteria and incorporating low-voltage mapping alongside advanced imaging techniques, such as LGE-CMR, into standardized ablation protocols. However, to achieve better clinical outcomes, ongoing refinements are required. For instance, while electro-anatomical mapping provides surrogate markers such as LVAs, these measures correlate inconsistently with histological findings and lack specificity for scar tissue, thereby limiting the efficacy of LVA-guided ablation. Similarly, LGE-CMR lacks standardization and sufficient spatial resolution to accurately quantify the extent of atrial fibrosis. Moreover, the high cost and limited availability of specialized centers and trained personnel restrict the accessibility of catheter ablation for many symptomatic AF patients worldwide. Lastly, it is important to note that catheter ablation does not address the underlying atrial cardiomyopathy that contributes to AF development.

Refining these aforementioned approaches and bridging existing knowledge gaps will require collaborative efforts across basic, translational, and clinical research domains.

## 9. Conclusions

The escalating global burden of both DM and AF has brought renewed focus to their complex, bidirectional interplay—an intersection now recognized as a critical frontier in cardiovascular research and clinical practice. In this review, we delineated the multifaceted pathophysiological mechanisms by which diabetes fosters an arrhythmogenic atrial substrate and highlighted emerging molecular targets and innovative rhythm control strategies poised to transform therapeutic paradigms.

Moving forward, the management of diabetes-associated AF demands a paradigm shift toward integrative and precision-guided approaches. This will necessitate the elucidation of targetable molecular pathways, the clinical translation of novel technologies—such as microRNA therapeutics, lipid nanoparticle-based drug delivery, and multi-omics biomarker discovery—and the refinement of diagnostic tools like low-voltage area (LVA) mapping and fibrosis quantification.

To establish the clinical efficacy of these strategies, robust validation through well-powered, multicenter randomized controlled trials is imperative. Ultimately, by closing current mechanistic gaps and embracing translational innovation, the field can advance toward a more tailored, disease-modifying framework for rhythm control—one that not only attenuates AF burden in patients with diabetes but also improves long-term cardiovascular outcomes at a population level.

## Figures and Tables

**Table 1:** Summary of promising drugs and investigational compounds for the treatment of diabetes-associated atrial fibrillation.

Compound	Target	Proposed Mechanism of Action	Major Effects
Anti-inflammatory compounds			
MCC950	NLRP3 inflammasome	Selective inhibitor of NLRP3 inflammasome by blocking ASC oligomerization	Alleviates fibrosis and AF in animal models [77].
Colchicine	NLRP3 inflammasome/ Chemotaxis	Inhibits β-tubulin polymerization into microtubules	Mixed evidence; COP-AF trial showed no significant AF reduction in patients who underwent major thoracic surgery [77,80].
CY-09	NLRP3 inflammasome	Inhibits NLRP3 oligomerization by binding to ATPase domain	Under investigation [77].
RRx-001	NLRP3 inflammasome	Inhibitor of NEK7 binding to LRR of the NLRP3 inflammasome	Under investigation [77].
JC-121, JC-124	NLRP3 inflammasome	Inhibitors of NLRP3 inflammasome activation and ASC aggregation	Under investigation [77].
Canakinumab	Pro-inflammatory cytokines	Monoclonal IL-1β neutralizing antibody	CONVERT-AF demonstrated a trend towards reduced AF-occurrence after cardioversion [82].
Pinocembrin	NF-κB	Abundant flavonoid suppresses inflammatory and fibrotic responses	Reduces atrial fibrosis, autonomic and electrical remodeling, and inflammation in MI animal model [83].
Tasquinimod	NF-κB	Inhibitor of the S100A8/A9 interaction and TLR4 signaling.	Enhances LV systolic function and suppresses pro-inflammatory cytokine expression in mice model [85].
Anti-fibrotic compounds			
Finerenone	TGF-β, Galectin-3, CTGF	Inhibit profibrotic signaling and atrial remodeling	It reduces risk of new-onset AF/AFL across the cardio–kidney–metabolic spectrum [95].
Macitentan	Endothelin-1	Endothelin-1 receptor antagonist	Decreased atrial endothelin 1 levels and prevented pacing-induced increases in pro-endothelin 1 mRNA [100].
Ion channel blockers/calcium handling modulators			
AVE0118	I_Kur_ current	I_Kur_, I_to_, and I_K,ACh_ blocker	Prolonged atrial refractory period without ventricular effects in experimental models [126,127,128,129].
XEN-D0101	I_Kur_ current	I_Kur_ blocker	Prolonged atrial refractory period without ventricular effects in experimental models [133,134].
KN-93 analogs	CaMKII, L-type calcium channels	Inhibits CaMKII and affects CaV1.3 and CaV1.2 calcium channels	It prevents pacing-induced AF in both in vitro and in vivo mouse models [154]
JTV519	RyR2 stabilizer	Inhibits I_K,Ach_ and I_Kur_ in atrial muscle cells	Under investigation [156].
Ranolazine	I_Na_,_L_ current	Selective inhibitor of I_Na_,_L_	Shown to reduce AF risk in clinical studies [135,136,137,138,139,140,141].
NCX modulators			
KBR7943	NCX	A selective NCX blocker; also inhibits I_to_, I_K1_, I_Na_, and I_Ca,L_.	Under investigation [150].
SEA-0400	NCX	A selective NCX blocker; additionally blocks I_Ca,L_	Under investigation [151].
Mitochondria-targeted antioxidants			
MitoQ	Mitochondria	Mitochondria-targeted antioxidant	Decreases aortic stiffness and improves endothelial function; mitigates atrial remodeling by neutralizing ROS [160].
Trimetazidine	Mitochondria	Metabolic modulator; activates mitochondrial complex I, promotes biogenesis, and reduces oxidative stress.	Prevents atrial structural remodeling [161].
KL1333	Mitochondria	NAD+ modulator, enhances the activity of AMPK/SIRT1/PGC-1a axis	Under investigation [162].
Glucose-lowering drugs			
SGLT2 inhibitors	SGLT2 protein	Exert antiarrhythmic effects via anti-inflammatory, antifibrotic, antioxidant, and electrophysiological modulation; enhance mitochondrial function, Ca^2+^ handling, and metabolic efficiency.	Significantly reduce AF risk and embolic stroke, independent of glycemic status. Reduction in atrial tachyarrhythmias in patients with cardiac implantable electronic devices [191,192,193,194].
GLP-1 RAs	Glucagon-like peptide-1 receptor	Exert antifibrotic, anti-inflammatory, antioxidant, and metabolic modulation; improve atrial electrophysiology and mitochondrial function.	Reduce major cardiovascular events and neutral AF risk overall, but may lower AF recurrence post-ablation; less effective than SGLT2is in AF prevention [195,196,210,211,212]
Metformin	Gluconeogenesis	Pleiotropic effects via antioxidant, anti-inflammatory, and AMPK-driven metabolic and electrophysiological modulation	Reduces AF burden [231,232,233,234].
TZDs	PPAR-γ receptors	Enhances insulin sensitivity via activation of PPAR-γ	Attenuates atrial fibrosis and shortens interatrial activation time, potentially decreasing AF susceptibility [235]

NLRP3, NOD-like receptor containing pyrin domain 3; ASC, apoptosis-associated speck-like protein; AF, atrial fibrillation; ATPase, adenosine triphosphatase; NEK7, NIMA-related kinase 7; LRR, leucine-rich repeat; MI, myocardial infarction; TLR4, Toll-like receptor 4; LV, left ventricle; TGFβ, transforming growth factor-β; CTGF, connective tissue growth factor; mRNA, messenger ribonucleic acid; AFL, atrial flutter; I_Kur_, ultrarapid delayed rectifier potassium current, I_to_, transient outward potassium current; I_K,Ach_, acetylcholine-activated potassium current; I_Na,L_, late sodium current; I_K1_, inward rectifier K^+^ current; I_Na_, sodium current; I_Ca,L_, L-type calcium current; CaMKII, Ca^2+^/calmodulin-dependent protein kinase II; RyR2, ryanodine receptor type 2 channel; NCX, Na^+^–Ca^2+^ exchanger; NF-κB, nuclear factor-κB; ROS, reactive oxygen species; NAD+, nicotinamide adenine dinucleotide; AMPK, activated protein kinase; SIRT1, sirtuin 1; PGC-1a, peroxisome proliferator-activated receptor-gamma coactivator-1alpha; SGLT2, sodium–glucose cotransporter 2; GLP-1 RAs, glucagon-like peptide-1 receptor agonists; TZDs, thiazolidinediones; PPAR-γ, peroxisome proliferator-activated receptor gamma.

## Data Availability

All data generated in this research is included within the article.
